# Molecular mechanism of empagliflozin cardioprotection in 5-fluorouracil (5-FU)-induced cardiotoxicity via modulation of SGLT2 and TNFα/TLR/NF-κB signaling pathway in rats

**DOI:** 10.1007/s43188-023-00204-1

**Published:** 2023-10-03

**Authors:** Marwa Monier Mahmoud Refaie, Sayed Shehata, Maram El-Hussieny, Michael Atef Fawzy, Nagwa Zenhom Mustafa Ahmed, Heba Marey, Asmaa Mohammed Hishmat, Turki Alkully, Eman Shaaban Mahmoud Abd El Rahman

**Affiliations:** 1 Department of Pharmacology, Faculty of Medicine, Minia University, El-Minia, 61511 Egypt; 2 Department of Cardiology, Faculty of Medicine, Minia University, El-Minia, 61511 Egypt; 3 Department of Pathology, Faculty of Medicine, Minia University, El-Minia, 61511 Egypt; 4 Department of Biochemistry, Faculty of Pharmacy, Minia University, El-Minia, 61511 Egypt; 5 Department of Biochemistry, Faculty of Medicine, Minia University, El-Minia, 61511 Egypt; 6 Department of Biochemistry, Faculty of Medicine, Al-Baha University, 65525 Albaha, Saudi Arabia; 7 Department of Forensic Medicine & Clinical Toxicology, Faculty of Medicine, Minia University, El-Minia, 61511 Egypt; 8 Department of Internal Medicine, Faculty of Medicine, Al-Baha University, 65525 Albaha, Saudi Arabia

**Keywords:** Empagliflozin, 5-Fluorouracil, Cardiotoxicity, Toll like receptor

## Abstract

One of the commoly used chemotherapeutic agents is 5-Fluorouracil (5-FU). Unfortunately, the clinical administration of 5-FU is complicated with serious cardiotoxic effects and the safe use becomes an urgent task in cardio-oncology. Till now, there are no studies discussed the role of empagliflozin (EMP) against 5-FU cardiotoxicity. Thus, we investigated this effect and the involved mechanisms in 5-FU induced heart injury. Forty male rats of Wistar albino species were used and divided randomly into four groups. Group I is the control group, group II is EMP given group, group III is 5-FU cardiotoxic group and group IV is 5-FU plus EMP group. 5-FU (150 mg/kg) was administered as a single intraperitoneal (i.p.) dose on 1st day to induce cardiotoxicity with or without EMP (30 mg/kg/d) orally for 5 days. The dose of 5-FU is relevant to the human toxic dose. Our data showed that 5-FU given group caused cardiotoxicity with significant increase of serum cardiac enzymes, toll like receptors, enhancement of nuclear factor kappa B (NF-κB), interleukin1β (IL1β), IL6, myeloid-differentiation-factor 88 (MYD88), heart weight, malondialdehyde (MDA), tumor-necrosis-factor-alpha (TNFα), sodium glucose co-transporter 2 (SGLT2), P53 and caspase3 expression with clear histopathological features of cardiotoxicity. Moreover, there is a significant decrease in reduced glutathione (GSH) and total antioxidant capacity (TAC). Interestingly, co-administration of EMP could ameliorate 5-FU induced biochemical and histopathological changes. This effect may be due to modulation of SGLT2, decreasing inflammation, oxidative stress and apoptosis with downregulation of an essential inflammatory cascade that mediates 5-FU cardiotoxicity; TNFα/TLR/NF-κB.

**Supplementary Information:**

The online version contains supplementary material available at 10.1007/s43188-023-00204-1.

## Introduction

One of the widely used thymidylate synthase inhibitors is 5-fluorouracil (5-FU) which is included in different regimens of cancer treatment such as colorectal cancer, squamous cell carcinoma and renal cell carcinoma. Unfortunately, 5-FU is considered as the second most commonly used cardiotoxic drug leading to myocardial ischemia, infarction, arrhythmias, myocarditis and heart failure or even death. Different pathways mediate 5-FU cardiotoxicity such as oxidative stress accompanied with release of reactive-oxygen-species-(ROS) [1, 2]. However, cardiac tissue has a minimal ability to act against their harmful effects because of low level of antioxidant enzymes. Furthermore, 5-FU causes direct cardiac damage and increases the rate of oxygen consumption. This occurs due to mitochondrial uncoupling, injury of the endothelium with depletion of endothelial nitric oxide synthase leading to vasospasm then ischemic injury [2–4].

The early response of the cardiac tissue to oxidative injury is the activation of tumor necrosis factor alpha (TNFα) and nuclear factor-κB (NF-κB) followed by stimulation of toll like receptors (TLR) that induce apoptosis and cell death upon activation of several caspases [2, 5]. In addition, TLRs control several pathways in innate immunity and stimulation of these receptors could increase the gene expression of other inflammatory mediators as myeloid differentiation factor 88 (MYD88) signaling cascade [6, 7]. Moreover, NF-κB has an ability to activate macrophages including (M1) and (M2). M1 produces the pro-inflammatory cytokines, such as IL-1 and TNF-α. In contrast, M2 releases the anti-inflammatory ones including IL-10 and IL-13 [5]. There is an evidence suggests that MyD88/TLR pathway is a crucial factor for M1 macrophage polarization and enhancing the expression of pro-inflammatory cytokines [5, 8]. Release of interleukins and the family-related proteins is a key factor in mediating cardiotoxic injury of anticancer drugs including IL1β, IL6. Thus, controlling the production of these agents represents an essential strategy in treatment of 5-FU-induced heart damage [3]. Furthermore, SGLT are widely distributed in the myocardium and upregulation of them leads to cardiac remodeling, hypertrophy, induce different inflammatory pathways and fibrogenesis so that modulation of these recptors is a critical target to rescue the myocardium [9].

Sodium glucose co-transporter inhibitors (SGLTI) including empagliflozin (EMP) are efficacious oral anti-diabetic drugs. The beneficial role of this drug group is based on decreasing blood glucose level and preventing renal tubular glucose reabsorption in the proximal-convoluted-tubules [10, 11]. Furthermore, it diminishes the inflammatory cytokines with an ability to scavenge reactive oxygen and nitrogen species and decrease cardiac inflammation [12–14], control renin angiotensin aldosterone system, ameliorate renal and cardiac complication in diabetic patients and doxorubicin cardiotoxicity. Moreover, EMP enhances NO release, induces vasodiltation and improves endothelial function that could counteract the damaging effect of anticancer drug [15–17].

Depending on EMP pharmacological properties, the pathogenesis of 5-FU cardiotoxic effect and the critical need to discover more guarding agents against 5-FU harmful effects, we aimed to evaluate the ability of EMP to control such toxicity based on modulating SGLT with studying the involved mechanisms focusing on TNFα/TLR/NF-κB/MYD88 pathway.

## Materials and methods

### Ethical approval

This study followed the guidelines for the care of experimental animals and it received the approval of the Institutional Ethical Committee, Faculty of Medicine, Minia University, Egypt based on ARRIVE guidelines for taking care and use of laboratory animals, EU Directive 2010/63/EU for animal experiments, the National Research Council’s Guide for the Care and Use of Laboratory. Approval Number: 331-4-2022.

### Chemicals

5-FU was obtained from Hikma Pharmaceutical Co. (6th of October, Egypt). EMP was from Boehringer Ingelheim co. Germany. Elisa kits for measuring cardiac enzymes including creatine kinase-MB (CK-MB) (Catalog # MBS2515061), troponin I (Catalog # MBS722833), lactate dehydrogenase (LDH) (Catalog # MBS043166), SGLT2 (Catalog # MBS1600381), P53 (Catalog # MBS723886), TNFα (Catalog # MBS2507393), TLR4 (Catalog # MBS2024497) and NF-κB (Catalog # MBS453975) were purchased from My BioSource Co., San Diego, CA, USA. Total antioxidant capacity kit (TAC) (Catalog # TA2513) was from Biodiagnostic, Egypt. The polyclonal rabbit/anti-rat MYD88 (Catalog # PA5-19918) and TLR5 (Catalog # PA1-41139) antibodies and the immunostaining detection kits were from Thermo Fisher Scientific Inc. Total Protein-Thiol-Assay Colorimetric-Kit (ab219272, Abcam).

### Study protocol

Forty male rats of Wistar albino species weighed about 200–220 g were purchased from Animal Research Centre, Giza, Egypt. They were kept in a suitable housing condition (3 rats/cage) and left to acclimatize one week with free access to both chow and tap water. Rats were divided randomly into 4 groups (n = 10) and EMP was dissolved in 1% carboxymethylcellulose and 5-FU was dissolved in saline just before administration. Dose of 5-FU was detected according to the previous studies and our pilot study which is relevant to the toxic dose in human. The preliminary part of our study was repeated for 3 times to ensure the success of our model.

**Group Ӏ**: vehicle (1% carboxymethylcellulose) was given orally by gavage for 5 days.

**Group II:** EMP (30 mg/kg/day) [18] was administered orally by gavage for 5 days.

**Group III**: 5-FU (150 mg/kg) single intraperitoneal (i.p.) dose in 1st day [19] plus vehicle were given orally by gavage for 5 days.

**Group IV:** 5-FU (150 mg/kg) single i.p. dose in 1st day [19] plus EMP (30 mg/kg/d) [18] were given orally by gavage for 5 days.

### Detection of blood pressure

Blood pressure (BP) was detected before animal scarifice using tail-cuff method (LETICA, Panlab S.L., Barcelona, Spain). Each rat was kept calm at 38 ℃ for 15 min for relaxation then we detected the rat tail artery pulsation and the tail–cuff was applied and BP was measured for five successive times in each animal. Results were based on the mean of the several successive measurements [20].

### Samples and their storage

On 5th day, each animal was anesthetized by i.p. injection of a general anesthetic agent 20% Urethane hydrochloride (1gm/kg). Arterial blood sample of each rat was obtained from abdominal aorta then centrifuged for 15 min at 5000 rpm to separate the clear sera (JanetzkiT30 centrifuge, Germany). The heart of each rat was excised, washed and weighed. Part of each ventricle was fixed in 10% formalin then embedded in paraffin for histopathological and immunohistochemical evaluation. Another part was stored at − 80 ℃ for further preparation of homogenates in phosphate buffer saline 20%w/v by GLAS-Col homogenizer, USA with centrifugation for 20 min at 3000 rpm then separating the supernatant of each sample and aliquots were kept at − 80 ℃ untill used.

### Measurement of NF-κB, TLR4, TNFα, SGLT-2, P53 and serum cardiac enzymes

We measured cardiac enzymes, NF-κB, TLR4, TNFα, SGLT-2 and P53 by using commercial ELISA kits based on the manufacturers’ instructions depending on sandwich ELISA immunoassay technique. The microtiter plate of each measurement was pre-coated with a monoclonal antibody that is specific for each one then we added the standards or the samples to the microtiter plate wells. The detected protien bound to the antibody pre-coated wells. The reaction was terminated by adding sulphuric acid solution and the change of color was measured spectrophotometrically at the specific wave length of each parameter. The color intensity was detected in proportional to the concentration of the measured protien.

### Evaluation of oxidative stress parameters

Serum TAC was measured at 510 nm by using colorimetric kit which was purchased from Biodiagnostic, Egypt and the result was expressed in mmol/ml. Tissue MDA was evaluated colorimetrically at 535 nm and results were expressed in mmol/g protien using 1, 1, 3, 3-tetramethoxypropane standard curve [21]. Tissue GSH evaluation was based on the binding of sulfhydryl group with Ellman’s reagent and formation of a yellow color measured colorimetrically at 405 nm by Beckman DU-64 UV/VIS spectrophotometer, USA in a unit of mmol/g protien [22].

### Western blotting measurement of caspase 3, IL1β, IL6, TLR2

Total Protein was measure by Thiol Assay Colorimetric Kit (ab219272, Abcam). Fifty μg of protein was obtained from heart tissue homogenates which were boiled for 5 min in a buffer containing 2-mercaptoethanol followed by loading on 12% sodium dodecyl sulfate–polyacrylamide gel electrophoresis and running for 2 h at 100 V. After electrophoresis, proteins were blotted to polyvinylideneflouride membranes. Blocking step for 1 h in a tris-buffered saline was applied to the samples and a blocking solution contained 5% (w/v) non-fat milk and 0.05% Tween-20. Overnight incubation was at 4 ℃ with primary antibodies (1:1000) for rabbit anti-caspase 3 (ab214430, Abcam, Cambridge, UK), anti-IL1β (ab283818, Abcam, Cambridge, UK), anti-IL6 (ab9324, abcam, Cambridge, UK), anti-TLR2 (ab209217, Abcam, Cambridge, UK) and anti-(Glyceraldehyde-3-phosphate dehydrogenase) β-Actin antibody were allowed overnight at 4 ℃. Goat anti-rabbit polyclonal immunoglobulin conjugated with horseradish peroxidase (Cell Signaling Technology Inc., MA, USA) was used as a secondary antibody (1:5000) in blocking buffer. Bands were visualized by chemiluminescence, using an enhanced chemiluminescence kit (ECL, GE Healthcare, Chicago, IL, USA. Protein bands were evaluated densitometrically relative to β-Actin arbitrary units using Image J Software [23, 24].

### Histopathological evaluation

After sacrifice, part of every heart ventricle was dissected, fixed 10% formalin solution for 24 h then processed to prepare paraffin blocks which were cut into 4 μm sections for hematoxylin and eosin stainning and immunohistochemistry. The pathologist conducted the evaluation process of these slides in a blinded fashion to different groups using light microscopy (Olympus microscope, Japan). Scoring of the histopathological abnormalitiess was performed and the following changes were evaluated; the degree of disruption of cardiac muscles architecture, vascular congestion, inflammatory infiltrate, and necrosis. The slides were graded semi quantitatively: score ( −) = no changes, score ( +) = mild changes, score (+ +) = moderate and score (+ + +) = severe changes [25].

### Immunohistochemical procedure

Briefly, slides were de-paraffinized with xylene, hydrated through gradient ethyl alcohol then treated with 3% hydrogen peroxide for 30 min. Slides were washed in phosphate buffer saline and boiled for 15 min in a citrate buffer (pH 6.0) by microwave then cooled to room temperature. The primary MYD88 (1:100) and TLR5 (1:100) antibodies were applied and incubated overnight in a humidity chamber. Then these slides were washed in phosphate buffer saline before applying the biotinylated secondary antibody for 30 min and streptavidin–biotin complex reagent for another 30 min. Afterwards; the 3, 3-diaminobenzidinetetra hydrochloride was applied and counterstained with hematoxylin and covered.

Regarding immunostaining of both MYD88 and TLR5; intensity was scored as follows: 0 = negative; 1 = weak; 2 = moderate; and 3 = strong staining. The positively stained area was scored as 0–100%. The final immunohistochemical score was got by multiplication of the intensity- score times the percentage of positively stained celluar area, resulting in values ranging from 0 to 300 [26, 27].

### Statistical analysis

Data of our study were analyzed by one way ANOVA followed by the Tukey’s multiple comparison test. Our results were expressed as means ± SEM and for statistical analysis; GraphPad Prism software program (version 5) was used. The differences were considered as significant results when the *p* value less than 0.05.

## Results

### Effect of EMP on heart weight, blood pressure and serum cardiac enzymes levels (troponin I, CK-MB, LDH)

5-FU (150 mg/kg) single toxic dose led to significant increase of heart weight, blood pressure and cardiac enzymes in comparison to control group and 5-FU treated group. However, co-administration of EMP diminished them significantly compared to 5-FU given group alone (Table 1).

**Table 1:** Effect of EMP on serum cardiac enzymes, blood pressure, heart weights

Groups	CK-MB (U/L)	LDH (U/L)	Troponin I (U/L)	BP(mmHg)	Heart weight/mg
CON	203.9 ± 4.9	150.6 ± 2.8	10.2 ± 0.5	102.9 ± 2.7	299.6 ± 10.6
EMP	204.3 ± 4.9	164.5 ± 3.8	10.6 ± 0.9	107.5 ± 2.0	334.5 ± 12.9
5-FU	304.5 ± 9.0 ^a,c^	271.1 ± 5.7^a,c^	74.4 ± 4.4^a,c^	160.5 ± 4.1^a,c^	440.4 ± 18.2^a,c^
5-FU + EMP	250.0 ± 9.2^b^	189.6 ± 6.1^b^	25.8 + 1.4^b^	134.4 ± 4.9^ab^	348.4 ± 22.3^b^

Values represents means ± SEM of 10 animals in each group

It is considered significantly different if *p* value less than 0.05

CON represents control group, EMP is empagliflozin group, 5-FU represents 5-fluorouracil group, 5-FU + EMP is 5-fluorouracil plus empagliflozin group

CK-MB is creatine kinase MB, LDH is lactate dehydrogenase, BP is blood pressure

^a^Significant difference if compared to control

^b^Significant difference in comparison to 5-fluorouracil group

^c^Significant difference compared to 5-fluorouracil treated group

### Effect of EMP on MDA, GSH, and TAC in cardiac tissue

5-FU given group showed significant elevation in the tissue level of MDA but decrease in GSH, and TAC in comparison to control group and 5-FU treated group. On the contrary, EMP plus 5-FU given group showed a significant decrease of MDA level but increase of GSH and TAC in comparison to 5-FU given group (Table 2).

**Table 2:** Effect of EMP on MDA, GSH, TAC

Groups	MDA (mmol/g protien)	GSH (mmol/g protien)	TAC (mmol/ml)
CON	4.1 ± 0.3	7.4 ± 0.3	0.9 ± 0.03
EMP	5.0 ± 0.2	7.2 ± 0.3	0.7 ± 0.04
5-FU	11.4 ± 0.7^a,c^	1.8 ± 1.0^a^^,c^	0.5 ± 0.02^a,c^
5-FU + EMP	8.0 ± 0.6^b^	6.2 ± 0.3^b^	0.7 ± 0.02^ab^

Values represents means ± SEM of 10 animals in each group

It is considered significantly different if *p* value less than 0.05

CON represents control group, EMP is empagliflozin group, 5-FU represents 5-fluorouracil group, 5-FU + EMP is 5-fluorouracil plus empagliflozin group

MDA is malondialdehyde, GSH is reduced glutathione, TAC is total antioxidant capacity

^a^Significant difference if compared to control

^b^Significant difference in comparison to 5-fluorouracil group

^c^Significant difference compared to 5-fluorouracil treated group

### Effect of EMP on NF-κB, TNFα, TLR4, SGLT-2 and P53

NF-κB, TNFα, TLR4, SGLT-2 and P53 increased significantly in 5-FU given group compared to control group and 5-FU treated group. However, EMP plus 5-FU given group significantly decreased all of these parameters in comparison to 5-FU given group (Table 3).

**Table 3:** Effect of EMP on NF-κB, TNFα, TLR4 and SGLT-2

Groups	TLR4 (ng/g protien)	TNFα (ng/g protien)	SGLT-2 (ng/g protien)	NF-κB (ng/g protien)	P53 (ng/g protien)
CON	101.5 ± 2.0	109 ± 3.2	233.5 ± 13.4	10.0 ± 0.4	9.2 ± 8.0
EMP	105.5 ± 1.4	121.6 ± 2.1	260.5 ± 13.5	9.2 ± 0.6	13.3 ± 1.3
5-FU	172.1 ± 6.2^ac^	163.3 ± 5.4^ac^	630.0 ± 21.0^ac^	43.9 ± 2.4^ac^	71.6 ± 4.4^ac^
5-FU + EMP	119.8 ± 2.3^ab^	122.3 ± 1.5^ab^	491.0 ± 21.1^ab^	27.40 ± 1.6^ab^	31.9 ± 2.6^ab^

Values represents means ± SEM of 10 animals in each group

It is considered significantly different if *p* value less than 0.05

CON represents control group, EMP is empagliflozin group, 5-FU represents 5-fluorouracil group, 5-FU + EMP is 5-fluorouracil plus empagliflozin group

TNFα is tumor necrosis factor alpha, TLR4 is toll like receptor 4, SGLT2 is sodium glucose co-transporter 2, NF-κB is nuclear factor kappa B

^a^Significant difference if compared to control

^b^Significant difference in comparison to 5-fluorouracil group

^c^Significant difference compared to 5-fluorouracil treated group

### Effect of EMP on cardiac tissue level of caspase 3, IL1β, IL6, TLR2 expression by western blotting

**Fig. 1:**
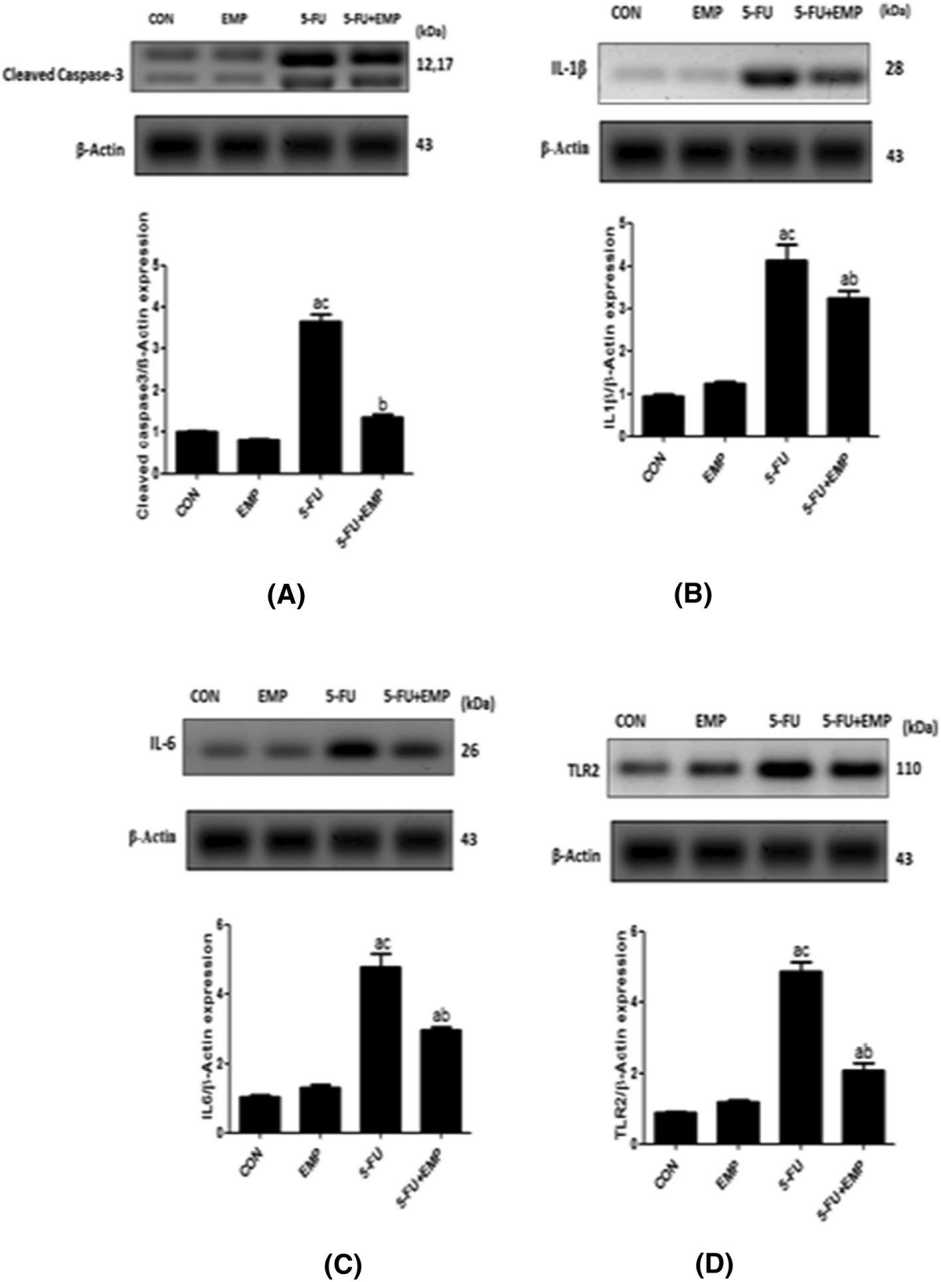
**a**–**d** Western blotting evaluation of caspase3, IL1β, IL6, TLR2 expression. Our data found significant increase of caspase3, IL1β, IL6, TLR2 expression in 5-fluorouracil (5-FU) given group compared to control group. However, empagliflozin (EMP) administration plus 5-fluorouracil (5-FU) revealed significant decrease of these parameters in comparison to 5-fluorouracil (5-FU) given rats. Values represents means ± SEM of 10 animals in each group. It is considered significantly different if *p* value less than 0.05. ^a^Significant difference if compared to control, ^b^significant difference in comparison to 5-fluorouracil group, ^c^significant difference compared to 5-fluorouracil treated group. CON represents control group, EMP is empagliflozin group, 5-FU represents 5-fluorouracil group, 5-FU + EMP is 5-fluorouracil plus empagliflozin group. IL1β is interleukin 1β, IL6 is interleukin 6, TLR2 is toll like receptor 2

Western blotting evaluation of caspase3, IL1β, TLR2, IL6 showed significant increase of their expressions in 5-FU given group compared to control group and 5-FU treated group. However, EMP co-administered group could significantly decrease caspase3, IL1β, IL6, TLR2 if compared to 5-FU untreated group (Fig. 1a–d).

### Histopathological evaluation results (Fig. 2)

**Fig. 2:**
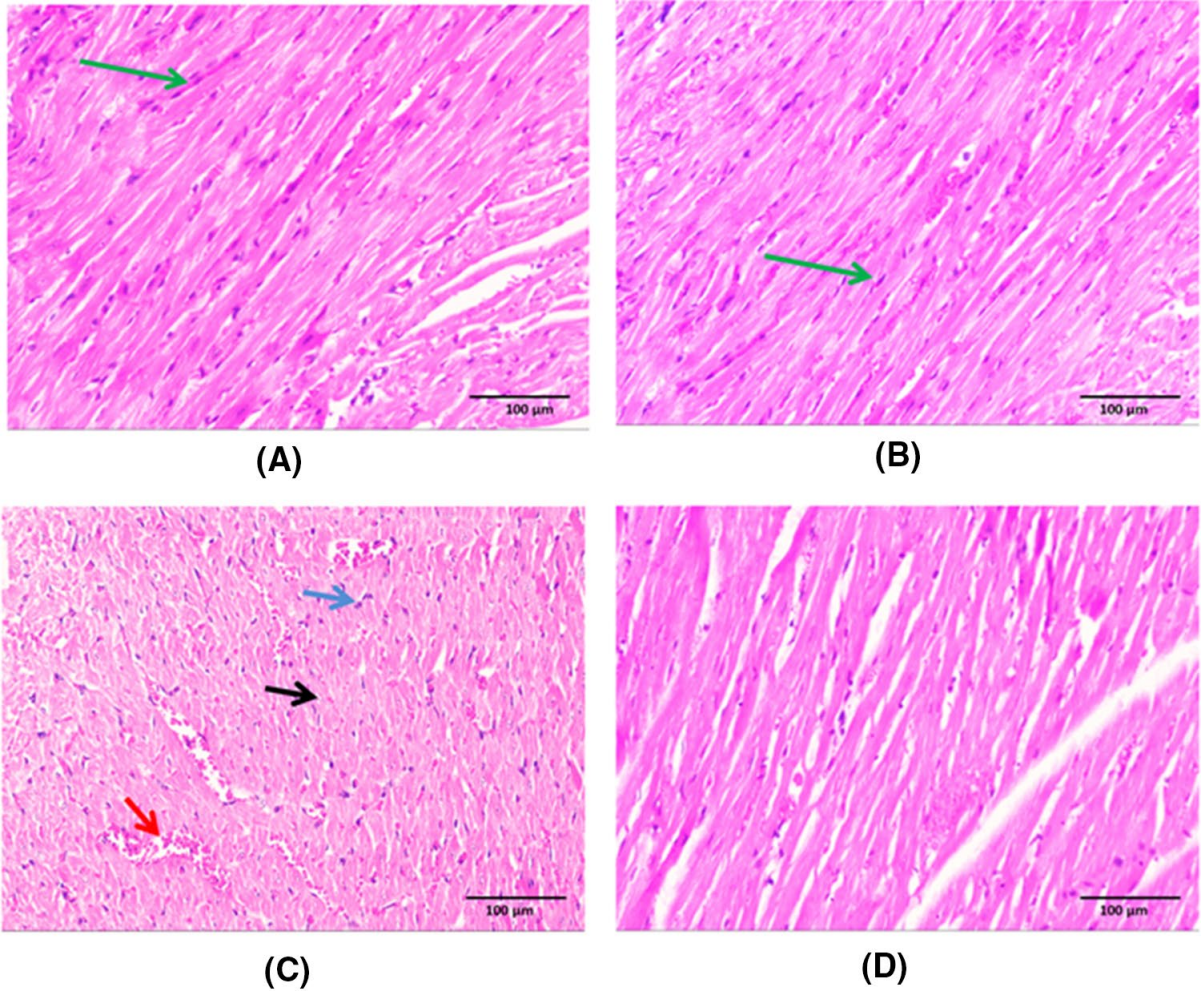
Histopathological evaluation results. Sections of both control group and empagliflozin (EMP) given group revealed preserved integrity of striated cardiac muscle fibres having acidophilic cytoplasm with central oval nuclei (green arrow) (**a** & **b**). The 5-fluorouracil (5-FU) cardiotoxic group showed disruption of architecture with loss of cardiac muscle striations, areas of necrotic cardiac tissue (black arrow), congested dilated blood vessels (red arrow) and inflammatory cellular infiltration (blue arrow) (**c**). There is significant improvement of the histopathological abnormalities observed in empagliflozin (EMP) co-administered group with restoration of cardiac muscle integrity (**d**). (X200). (Scale bar = 100 µm)

Sections of both control group and EMP given group revealed preserved integrity of the striated cardiac muscle fibres having acidophilic cytoplasm with central oval nuclei (a & b) respectively. 5-FU cardiotoxic group showed disruption of architecture with loss of cardiac muscle striations, areas of necrotic cardiac tissue, congested dilated blood vessels and inflammatory cellular infiltration (c). Marked amiloration of the histopathological abnormalities was observed in EMP co-administered group with restoration of cardiac muscle integrity (d). These data was supported by scoring of the histopathological findings (Table 4).

**Table 4:** Scoring of the histopathological abnormalitiess

Histopathological changes	CON	EMP	5-FU	5-FU + EMP
Disruption of cardiac muscles architecture	−	−	+ + +	+
Vascular congestion	−	−	+ +	+ +
Inflammatory cellular infiltrate	−	+	+ + +	+ +
Necrosis	−	−	+ + +	+

Scoring of the histopathological changes in different groups

The structural changes of tissue were assessed according to the degree of disruption of cardiac muscles architecture; vascular congestion; inflammatory cellular infiltrate and necrosis

Score ( −) = no changes, score ( +) = mild changes, score (+ +) = moderate and score (+ + +) = severe changes

CON is control group, EMP is empagliflozin group, 5-FU is 5-fluorouracil group, 5-FU + EMP is 5-fluorouracil plus empagliflozin group

### Evaluation of MYD88 immunoreactivity (Fig. 3)

**Fig. 3:**
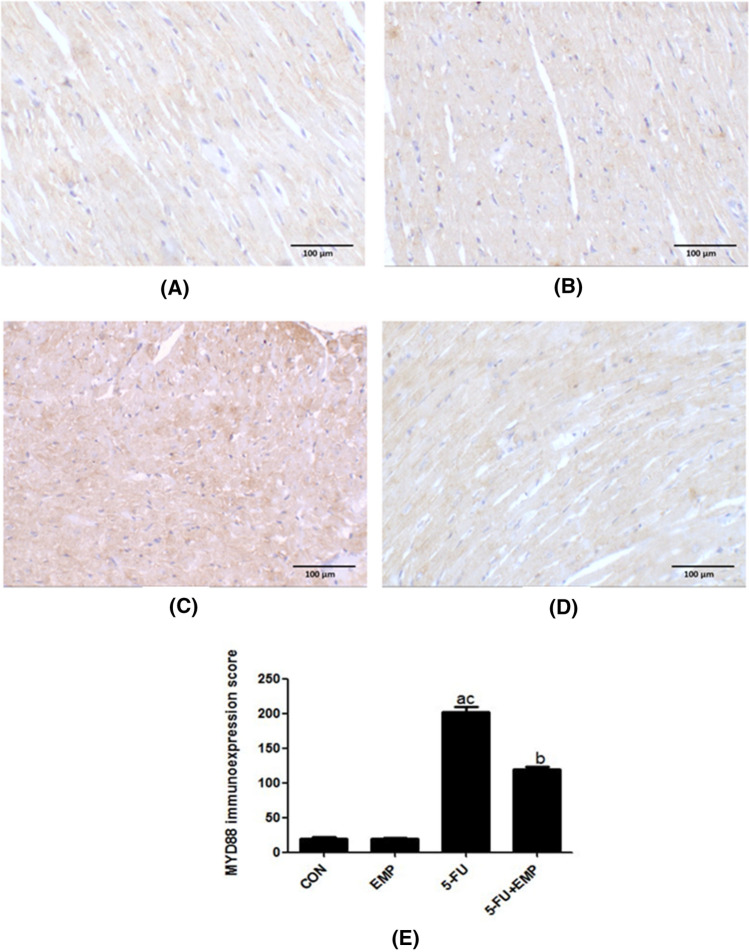
Evaluation of MYD88 immunoreactivity. Sections of both control and empagliflozin (EMP) given group showed weak positivity in the cytoplasm of cardiac muscles (**a**, **b**). Meanwhile, 5-fluorouracil (5-FU) administered group revealed strong immunoexpression in almost all the stained areas (**c**). Weak positivity was observed in the empagliflozin (EMP) co-administered group (**d**). (X200) (Scale bar = 100 µm). Semiquantitative analysis of MYD88 immunoexpression: Data revealed that the immunoexpression significantly increased in the 5-fluorouracil (5-FU) group compared to the control group. However, co-administration of empagliflozin (EMP) could significantly decrease its immunoexpression if compared to fluorouracil (5-FU) group (**e**). Values represents means ± SEM of 10 animals in each group. It is considered significantly different if *p* value less than 0.05. ^a^Significant difference if compared to control, ^b^significant difference in comparison to 5-fluorouracil group, ^c^significant difference compared to 5-fluorouracil treated group. CON represents control group, EMP is empagliflozin group, 5-FU represents 5-fluorouracil group, 5-FU + EMP is 5-fluorouracil plus empagliflozin group. MYD88 is myeloid differentiation factor 88

Control and EMP examined sections showed weak positivity in the cytoplasm of cardiac muscles (**a** & **b**). Meanwhile, 5-FU cardiotoxic group revealed strong immunoexpression in almost all the stained areas (**c**). Weak positivity was detected in the EMP co-administrated group (**d**) (Fig. 3).

### Semiquantitative analysis of cardiac tissue sections

Results revealed that MYD88 immunoexpression significantly increased in 5-FU untreated group compared to control group and EMP co-administrated group. However, EMP given group showed significant decrease of its immunoexpression compared to 5-FU given group (Fig. 3**e**).

### Evaluation of cardiac tissue TLR5 immunoreactivity (Fig. 4)

**Fig. 4:**
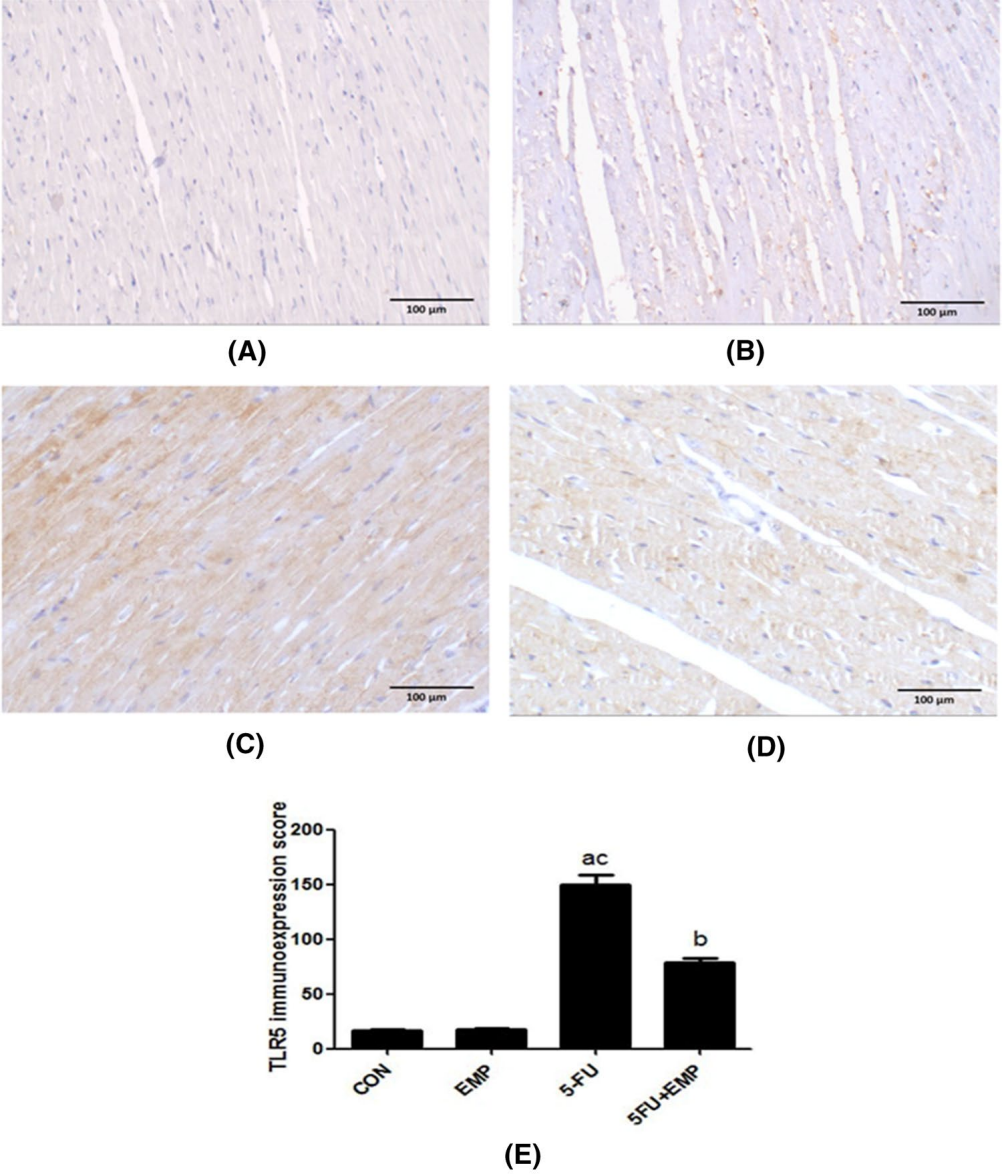
Evaluation of TLR5 immunoreactivity. Negative immunostaining was detected in both control and empagliflozin (EMP) given groups (**a**, **b**). On the other hand, the 5-fluorouracil (5-FU) given group showed strong positive immunoexpression (**c**). Meanwhile, the empagliflozin (EMP) co-administrated group exhibited weak immunostaining (**d**) (X200) (Scale bar = 100 µm). Semiquantitative analysis of TLR5 immunoexpression: Semiquantitative analysis showed that there is a significant increase of TLR5 immunoexpression in 5-fluorouracil (5-FU) group compared to control group. However, there is a significant decrease in its immunoexpression in empagliflozin (EMP) co-administered group compared to 5-fluorouracil (5-FU) group (**e**). Values represents means ± SEM of 10 animals in each group. It is considered significantly different if *p* value less than 0.05. ^a^Significant difference if compared to control, ^b^significant difference in comparison to 5-fluorouracil group, ^c^significant difference compared to 5-fluorouracil treated group. CON represents control group, EMP is empagliflozin group, 5-FU represents 5-fluorouracil group, 5-FU + EMP is 5-fluorouracil plus empagliflozin group. TLR5 is toll like receptor 5

Negative immunostaining was detected in both control and EMP given groups (**a**, **b**). On the other hand, 5-FU cardiotoxic group showed high positive immunoexpression (**c**). Meanwhile, the EMP co-administrated group exhibited weak immunostaining (**d**).

### Semiquantitative analysis of TLR5 immunoexpression in cardiac tissue sections

Results showed that there was a significant elevation of TLR5 immunoexpression in 5-FU group compared to both control group and 5-FU treated group. However, there was a significant decrease in its immunoexpression in EMP co-administered group compared to 5-FU untreated group (Fig. 4**e**).

## Discussion

Administration of 5-FU leads to serious cardiovascular toxicities associated with coronary spasm, angina, and myocardial infarction. Although the mechanisms of 5-FU harmful effect and the appropriate methods for preventing or treating its cardiovascular toxicities have not been clarified, it is clinicaly used for many years [1, 4]. This forced us to evaluate the possible protective properties of EMP in 5-FU induced cardiac damage. Our study found significant increases in the measured cardiac enzymes such as troponin I, CK-MB and LDH. Moreover, heart weights, blood pressure, SGLT-2, MDA, TLR2, TLR4, TLR5, MyD88, NF-κB, IL1β, IL6, P53 and caspase3 significantly elevated but GSH and TAC decreased with features of cardiotoxicity in the histopathological results in form of disturbed muscle striation, hemorrhage and inflammation. However, co-administration of EMP could ameliorate 5-FU induced changes with down-regulation of TNFα/TLR/NF-κB pathway, SGLT-2, oxidative stress, apoptosis and inflammation.

Several pathways are involved in mediating 5-FU induced cardiac injury including oxidative stress as the generation of free radicals enhances both intrinsic and extrinsic apoptotic pathways associated with activation of caspase family and p53 [28–31]. Furthermore, different cytokines such as IL1β, IL6 and TNFα can act on certain receptors and stimulate either cell death or survival. In addition, it is well known that the mitochondria-derived ROS could induce Ca^2+^ release from the endoplasmic reticulum ryanodine receptors initiating cellular necrosis and death [32, 33].

Antioxidants are considered the most significant defensive system to counteract oxidative stress and the associated mitochondrial dysfunction. There are non-enzymatic and enzymatic agents including glutathione, superoxide dismutase and catalase [34]. Our results are in line with previous studies which showed that 5-FU diminished the antioxidants as GSH and TAC but increased MDA which is considered as an essential indicator of both oxidative stress and membrane lipid peroxidation. Oxidative process damages the cardiac cell membrane and releases the intracellular cardiac enzymes outside the cell followed by elevation of their serum levels [35–37].

Another important signaling cascade in mediating 5-FU cardiotoxicity is TNFα/TLR/MyD88/NF-κB pathway. TLRs are a subfamily of recognizing receptors considered as a major regulator of both innate and adaptive immune response [32, 38]. In addition, TLRs have a great role in mediating different inflammatory and apoptotic disorders associated with releasing pro-inflammatory cytokines. One of TLR family is TLR5 which recognizes several pathogen-associated molecules leading to release of different inflammatory mediators and pro-fibrotic factors causing myocardial infarction. It has been approved that TLR5 deficiency could decrease inflammation, oxidative stress, attenuate cardiac fibrosis and dysfunction [39].

5FU upregulates inflammation and releases several pro-inflammatory agents leading to more and more activation of TLRs including TLR2, TLR4 that could initiate NF-κB pathway and form IL-1β, IL-6, TNFα [25, 40, 41]. The latter enhances programmed apoptotic process with an imbalance between both anti-apoptotic and pro-apoptotic factors upon releasing free radicals with elevation of P53 and several caspases including caspase3; a critical indicator of apoptosis [39, 41]. Our findings revealed significant increase and activation of TNFα/TLR/MYD88/NF-κB pathway in 5-FU administered group compared to control group and this is supported with previous studies [39–41].

Sodium glucose co-transporter 2 inhibitors (SGLTI2) including EMP; is a new class of anti-diabetic drugs used in type 2 diabetic patients and they act on proximal tubule and decrease glucose reabsorption. Despite their great benefit as cardioprotectant, still the mechanism of action is incompletely understood in different models [12, 16]. SGLT are highly expressed in the myocardium and they have a great role in mediating heart damage. EMP could modulate SGLT, ameliorate volume overload, decrease blood pressure and regulate renin angiotensin aldosterone system followed by decreasing cardiac remodeling and hypertrophy, attenuating inflammation and inhibiting the release of different pro-inflammatory cytokines accompanied with downregulation of apoptosis [9, 18, 42, 43].

EMP is able to ameliorate inflammation in different studies and this contributes to give cardiovascular benefits. These models demonstrated significant reductions of a large set of pro-inflammatory cytokines during administration of EMP including monocyte chemoattractant protein, IL6, P-selectin, TNFα, interferon, and intercellular adhesion molecule. Moreover, there are reductions in high sensitivity C-reactive protein and myeloperoxidase with increase in anti-inflammatory IL-10 [40–42].

Recently, it was found that the beneficial effect of SGLTI-2 as a cardioprotective agent may be caused by preventing sodium-hydrogen exchange in both kidneys and myocardium which is responsible for reuptake of sodium after filtration and it markedly increases in cardiac and renal patients [10, 44]. This abnormality may be responsible for the developed resistance to both endogenous natriuretic peptides and diuretics. Concerning EMP, it inhibits sodium-hydrogen exchange leading to reduction in heart injury, cardiac remodeling, hypertrophy, fibrosis, attenuation of myocardial dysfunction and natriuretic peptides resistance [15, 45]. This is confirmed in our results as we found that co-administration of EMP could decrease cardiac injury with marked improvement in both biochemical and histopathological changes. This cardiopreserving role of EMP was already detected in other models as diabetic cardiomyopathy, doxorubicin cardiotoxicity, and heart failure [9, 13, 45–49].

The ability to modify SGLT, regulation of renin angiotensin aldosterone system, anti-oxidant, anti-inflammatory and anti-apoptotic properties of EMP may be the suitable explanation of its protective effect in 5-FU induced cardiac injury [15–17, 48–50]. SGLT-2 is markedly involved in mediating heart damage thereby reducing its activity by EMP could decrease intracellular glucose and sodium, consequently 5’AMP-activated protein kinase that has a great role in cardiomyocyte injury. In addition, it was hypothesized that EMP could reduce interstitial fluid space, control cardiac congestion without reducing the arterial perfusion or filling leading to decrease the hospitalization of heart failure patients [13, 15, 42].

On the other hand, SGLTI-2 promote natriuresis, diuresis, reduce preload, decrease blood pressure, improve subendocardial blood flow without increasing heart rate. Furthermore, potent inhibition of SGLT2 prevents glucose and sodium reabsorption resulting in glycosuria and natriuresis without induction of hypoglycaemia [15, 51, 52]. According to our findings in Table3, there is increase in SGLT2 expression in 5-FU cardiotoxic group and decrease of its expression following EMP treatment. This reflects the essential role of SGLT2 in mediating 5-FU cardiotoxicity and EMP cardioprotective effect is dependent on SGLT2 modulation.

EMP has direct cardiac effects through regulating SGLT2 protein and its receptors. It could increase cell viability and preserve ATP levels following hypoxia/re-oxygenation. Moreover, EMP increases complex II respiration, improves mitochondrial function and cell viability. In addition, EMP changes the vascular smooth muscle cell function, induces vasodilation and increases blood follow [53, 54].

These pharmacological actions of EMP interfer with the harmful effect of 5-FU as the latter casuses coronary vasospasm and cardiac ischemia [55]. Current research paves the way to consider EMP as a cardioprotector to rescue the patient of 5-FU induced toxicity with myocardial damage.

EMP ameliorated 5-FU cardiotoxicity by different mechanisms including vasodilating effect, modulation of SGLT-2, anti-inflammatory, anti-apoptotic, anti-oxidant properties and inhibition of TNFα/TLR/MyD88/NF-κB pathway. More studies are recommended to evaluate the role of EMP in 5-FU cardiotoxic patients.

### Supplementary Information

Below is the link to the electronic supplementary material.

Supplementary file1 (PDF 227 KB)

## Data Availability

All data is available as supplementary material.
