# Supplementary material for: Molecular mechanism of empagliflozin cardioprotection in 5-fluorouracil (5-FU)-induced cardiotoxicity via modulation of SGLT2 and TNFα/TLR/NF-κB signaling pathway in rats
Source: Toxicol Res. 2023 Oct 3;40(1):139–51. doi: 10.1007/s43188-023-00204-1 (PMC10786789; doi:10.1007/s43188-023-00204-1)
Supplement: Supplementary file 1 — Supplementary file1 (PDF 227 KB) [file 43188_2023_204_MOESM1_ESM.pdf]

Interleukin 6 (IL-6)

Repeat 1(IL-6)

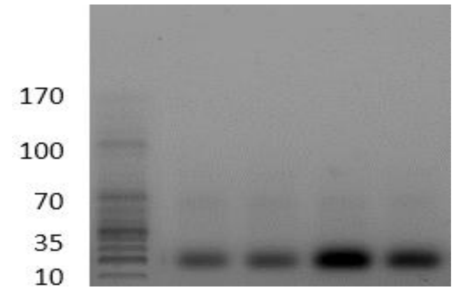

IL-6 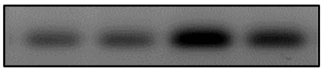 26 KDs

Repeat 2 (IL-6)

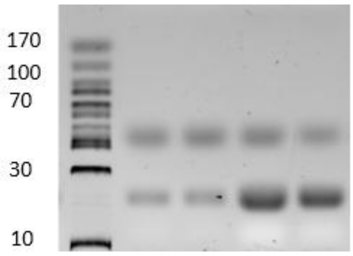

IL-6 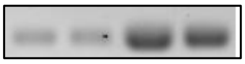 26 KDs

B-actin 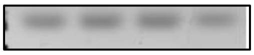 43 KD

Repeat 3 (IL-6)

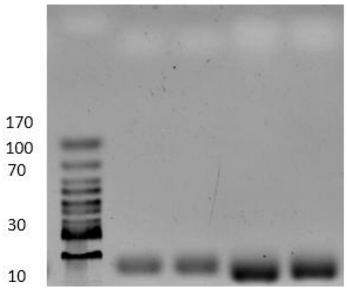

IL-6 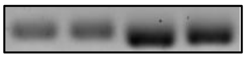 26 KDs

Pro Interleukin 1B (IL-1B)

Repeat 1

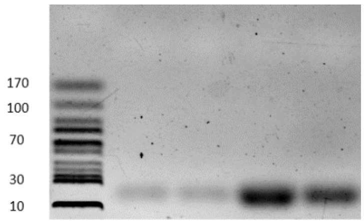

Pro IL 1B 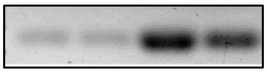 28 KDa

Repeat 2

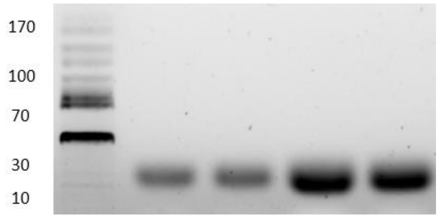

Pro IL 1B 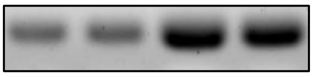 28 KDa

Repeat 3

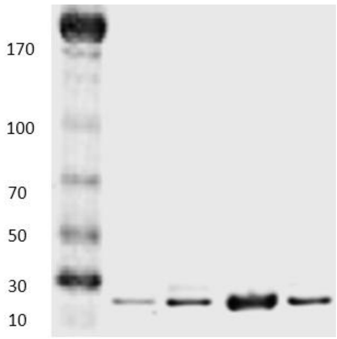

Pro IL 1B 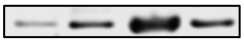 28 KDa

<u>TLR2</u>

Repeat 1

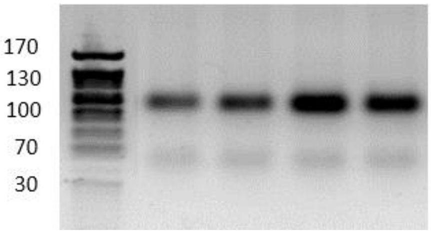

TLR2 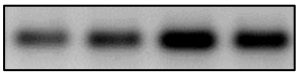 110 KDs

Repeat 2

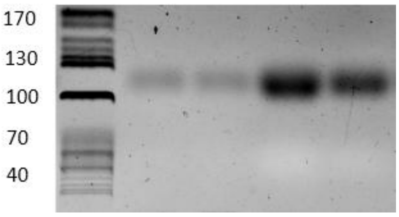

TLR2 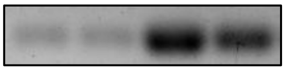 110 KDs

Repeat 3

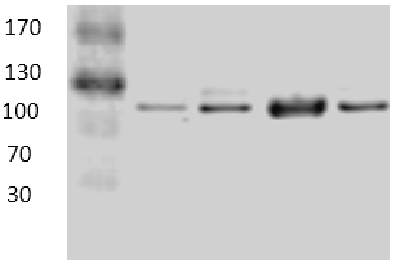

TLR2 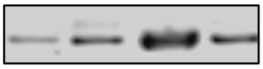 110 KDs

Cleaved Caspase 3

Repeat 1

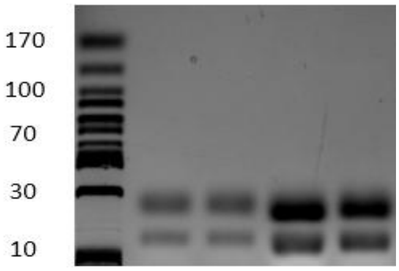

C-Caspase 3

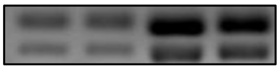

12,17 KDa

Repeat 2

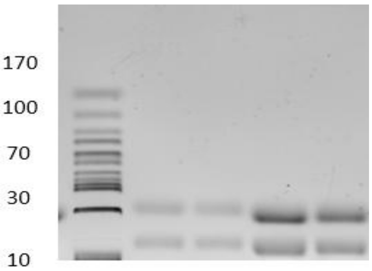

C-Caspase 3

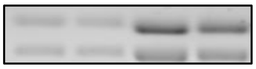

12,17 KDa

Repeat 3

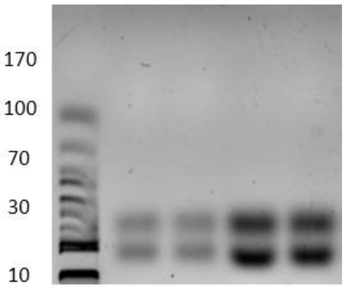

C-Caspase 3

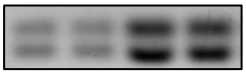

12,17 KDa

B-Actin

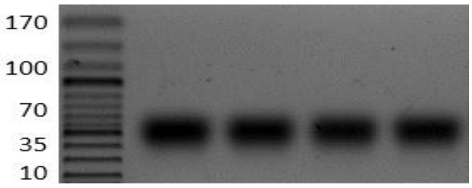

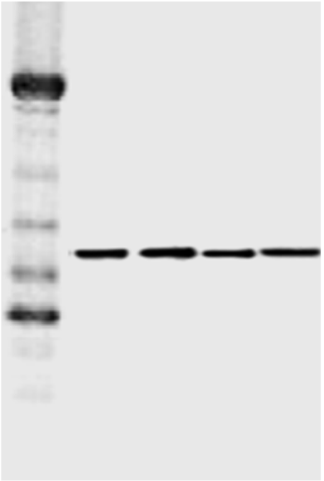

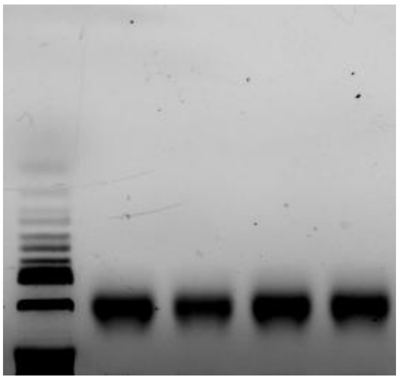

B-actin 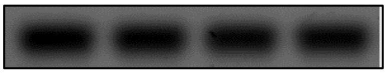 43 KDs
